# *EIF4G1* is a novel candidate gene associated with severe asthenozoospermia

**DOI:** 10.1002/mgg3.807

**Published:** 2019-07-03

**Authors:** Yanwei Sha, Wensheng Liu, Xianjing Huang, Yang Li, Zhiyong Ji, Libin Mei, Shaobin Lin, Shuangbo Kong, Jinhua Lu, Lingyuan Kong, Xingshen Zhu, Zhongxian Lu, Lu Ding

**Affiliations:** ^1^ Department of Andrology, United Diagnostic and Research Center for Clinical Genetics, School of Public Health & Women and Children's Hospital Xiamen University Xiamen China; ^2^ School of Pharmaceutical Sciences, State Key Laboratory of Cellular Stress Biology Xiamen University Xiamen China; ^3^ Fujian Provincial Key Laboratory of Reproductive Health Research Medical College of Xiamen University Xiamen China

**Keywords:** biallelic mutation, *EIF4G1*, severe asthenozoospermia, whole exome sequencing

## Abstract

**Background:**

Asthenozoospermia (AZS), also known as asthenospermia, is characterized by reduced motility of ejaculated spermatozoa and is detected in more than 40% of infertile patients. Because the proportion of progressive spermatozoa in severe AZS is <1%, severe AZS is an urgent challenge in reproductive medicine. Several genes have been reported to be relevant to severe asthenospermia. However, these gene mutations are found only in sporadic cases and can explain only a small fraction of severe AZS, so additional genetic pathogenies need to be explored.

**Methods and results:**

By screening the variant genes in a patient with severe AZS using whole exome sequencing, we identified biallelic mutations c.2521C>T: p.(Pro841Ser) (NC_000003.11: g.184043412C>T) in exon13 and c.2957C>G: p.(Ala986Gly) (NC_000003.11: g.184045117C>G) in exon17 in the eukaryotic translation initiation factor 4 gamma 1 gene (*EIF4G1*, RefSeq: NM_004953.4, OMIM: 600495) of the patient. Both of the mutation sites are rare and potentially deleterious. Transmission electron microscopy analysis showed a disrupted axonemal structure with mitochondrial sheath defects. The EIF4G1 protein level was extremely low, and the mitochondrial marker cytochrome c oxidase subunit 4I1 (COXIV, OMIM: 123864) and mitochondrially encoded ATP synthase 6 (ATP6, OMIM: 516060) protein levels were also decreased in the patient's spermatozoa as revealed by WB and IF analysis. This infertility associated with this condition was overcome by intracytoplasmic sperm injections, as his wife became pregnant successfully.

**Conclusion:**

Our experimental findings indicate that the EIF4G1 gene is a novel candidate gene that may be relevant to severe AZS.

## 1 INTRODUCTION

Infertility is a major public health issue that affects approximately 7%–12% of couples worldwide (Dirami et al., 2013). The motility of a spermatozoon is categorized as progressive motility (PR), nonprogressive motility (NP) or immotility (IM). A male has asthenozoospermia (AZS) when his total motility (PR + NP) is less than 40% or when his PR is below 32%. Asthenozoospermia is a common cause of infertility and can be detected in more than 40% of infertile patients (Dirami et al., 2013; Xu et al., 2018). Asthenospermia is divided into four types, namely, mild asthenozoospermia, moderate asthenozoospermia, severe asthenozoospermia, and complete asthenozoospermia, according to the degree of impairment of sperm motility (Al‐Malki et al., 2017; Amer, Metawae, Hosny, & Raef, 2013; Casper, Meriano, Jarvi, Cowan, & Lucato, 1996; Katsumi et al., 2014). Because the proportion of progressive spermatozoa in severe asthenozoospermia is <1%, severe asthenozoospermia is an urgent challenge in reproductive medicine. The mechanism of severe asthenozoospermia, including metabolic deficiencies (Wilton, Temple‐Smith, & de Kretser, 1992), tail anatomical abnormalities (Collodel et al., 2011; Moretti et al., 2011), genital tract infections (Lores et al., 2018), varicocele (Amer, Mostafa, Fathy, Saad, & Mostafa, 2015; Mostafa et al., 2018; Mostafa, Rashed, & Taymour, 2016), an unhealthy lifestyle, antisperm antibodies (Dimitrov et al., 1994; Harrison, 1978; Marchini et al., 1991; Shibahara, Shiraishi, & Suzuki, 2005) and necrozoospermia (Gopalkrishnan, Padwal, D'Souza, & Shah, 1995), is complex. However, the exact pathogenesis of severe asthenozoospermia remains largely unclear.

Previous studies have revealed that severe asthenozoospermia may be a genetic disease (Chemes et al., 1998; Collodel et al., 2011; Xu et al., 2018). Several genes, namely, sperm‐associated antigen 17 (*SPAG17*, OMIM: 616554) (Xu et al., 2018); A‐kinase anchoring protein 3 (*AKAP3*, OMIM: 604689) and A‐kinase anchoring protein 4 (*AKAP4*, OMIM: 300185; Baccetti et al., 2005); septin 4 (*SEPT4,* OMIM: 603696; Li et al., 2011); dynein axonemal heavy chain 1 (*DNAH1*, OMIM: 603332; Amiri‐Yekta et al., 2016; Ben Khelifa et al., 2014; Sha, Yang, et al., 2017; Wang et al., 2017); cation channel sperm associated 2 (*CATSPER2,* OMIM: 607249; Zhang et al., 2007); polypeptide N‐acetylgalactosaminyltransferase like 5 (*GALNTL5*, OMIM: 615133; Takasaki et al., 2014); and NOP2/Sun RNA methyltransferase family member 7 (*NSUN7,* OMIM: 617185; Khosronezhad, Colagar, & Jorsarayi, 2015), have been reported to be relevant to severe asthenospermia. These genes are associated with central pair microtubules, sperm midpiece defects, the axoneme, anion fluxes or glycolytic enzymes. However, mutations in these genes have been found only in sporadic cases and can explain the pathogenesis of only a small fraction of patients with severe asthenozoospermia. Therefore, further exploration is needed to uncover the pathogenesis of severe asthenozoospermia.

The *EIF4G1* (eukaryotic translation initiation factor 4G1) gene is approximately 20.8‐kb long and contains 31 exons encoding 1599 amino acids (Yan & Rhoads, 1995). EIF4G1 is a scaffold protein and recruits eIF4E and eIF4A to form an eIF4 complex, which regulates the translation initiation of mRNAs (Byrd, Zamora, & Lloyd, 2002; Chartier‐Harlin et al., 2011; Imataka & Sonenberg, 1997; Ramirez‐Valle, Braunstein, Zavadil, Formenti, & Schneider, 2008). The EIF4G1 expression level is significantly changed in poly(A)‐binding protein‐interacting protein 2 (Paip2, HGNC:17970) a/b double‐null mutant (DKO) mice. Paip2a plays an important role in translational control in late spermiogenesis. Both Paip2a null mice and Paip2a/Paip2b DKO mice are infertile, with defective sperm morphology (Delbes, Yanagiya, Sonenberg, & Robaire, 2012; Yanagiya, Delbes, Svitkin, Robaire, & Sonenberg, 2010). EIF4G1 is associated with Poly(A)‐binding protein cytoplasmic 1/2 (PABPC1, HGNC:8554), two important regulators in translational repression in the cytoplasm of meiotic and early haploid spermatogenic cells (Kimura, Ishida, Kashiwabara, & Baba, 2009). These findings suggest that *EIF4G1* may participate in the process of transcriptional regulation in late spermiogenesis in mice. However, the role of *EIF4G1* in late spermiogenesis, especially in human sperm, remains largely unclear. Thus, more research is needed to determine the role of the EIF4G1 gene in reproduction.

Here, we found biallelic mutations in *EIF4G1* from an infertile patient with severe asthenozoospermia and revealed that these mutations may be the key factor responsible for infertility due to this condition.

## 2 MATERIALS AND METHODS

### 2.1 Patient and control subject

The proband (30 years of age, II:4) was recruited from the Xiamen Maternity and Child Care Hospital. He was healthy and had a normal sexual ability, but he has not been able to make his wife pregnant. His testes and accessory glands were normal; the reproductive hormones were in normal ranges (FSH 2.25 mIU/mI, LH 1.92 mIU/ml, T 3.94 ng/ml, E2 27 pg/ml, and PRL 7.01 ng/ml), and the biochemical test indexes of the seminal plasma were normal. The semen examination results were as follows: semen volume, 2.1 ml; semen pH, 7.2; sperm density, 58.8 million/ml; percentage of motile sperm, 11.3%; and percentage of progressive sperm, 0.2%. Sperm morphological analysis showed that the normal sperm morphology amounted to 6%. The chromosomal karyotype of the patient was normal: 46, XY. No microdeletions were found in the Y chromosome. Based on these results, the patient was diagnosed with severe asthenozoospermia. Five milliliters of peripheral blood was collected from the patient, his elder brother and his parents. The control subject was a healthy male aged 28 with normal fertility.

### 2.2 Ethical compliance

This study was approved by the Ethics Committee of Xiamen Maternity and Child Care Hospital. Written informed consent was obtained from each participant.

### 2.3 Whole exome sequencing and sanger sequencing validation

Whole exome sequencing (WES) was performed as described previously (Sha et al., 2018). Briefly, a DNA library was prepared, and exomes were concentrated with the TruSeq Exome Enrichment kit (Illumina, San Diego, CA) following the manufacturer's protocol. Then, high‐throughput sequencing was performed using the Illumina Hiseq 2000 sequencer. The reads were aligned against UCSC hg19 with the Burrows‐Wheeler Aligner (http://biobwa.sourceforge.net/). Variants were annotated by the ANNOVAR (http://www.openbioinformatics.org/annovar/) database, Mutation Taster (http://www.mutationtaster.org/) database, the Exome Aggregation Consortium (ExAC) (http://exac.broadinstitute.org/), and the 1,000 Genomes project (http://www.1000genomes.org/data) databases for pathopoiesia, novelty, and frequency. Mutations that met the following criteria were retained for subsequent analyses: missense, nonsense, frame‐shift, or splice site variants and variants that were absent or rare (variants with a minor allele frequency <1% in the ExAC and 1,000 Genomes datasets by considering the rare prevalence of severe asthenozoospermia). Sanger sequencing was used to validate the mutation of the *EIF4G1* gene in the patient and his parents. The primers used for Sanger sequencing are listed in Table S1.

### 2.4 Papanicolaou staining

Papanicolaou staining of the spermatozoa was performed according to the World Health Organization standards for human semen examination and processing (5th ed.) with modification to confirm morphological changes in sperm tails as described previously (Sha, Yang, et al., 2017). Briefly, slides were fixed in 95% ethanol for 15 min, followed by immersion in a graded alcohol series from 80% to 50%. Then, the slides were rinsed with distilled water for 2 min and stained with hematoxylin for 5 min. After soaking with distilled water and ethanol hydrochloride, the slides were inserted into Bluing Reagent for 4 min. Then, the slides were dehydrated in a graded alcohol series from 50% to 90% and dyed with Orange G6 and EA50. Then, the slides were dehydrated with 95% and absolute alcohol. Subsequently, slides were washed in xylene and mounted with permanent mounting medium.

### 2.5 Transmission electron microscopy

Sperm samples were examined following a previously published procedure for subcellular structural changes in sperm (Sha, Yang, et al., 2017). Briefly, prepared spermatozoa were immobilized with 2.5% phosphate‐buffered glutaraldehyde. Then, the samples were washed with 0.1 M phosphate buffer (pH 7.2) three times and postfixed with 1% osmium tetroxide. Dehydration was performed using a graded alcohol series and 100% acetone sequentially, followed by infiltration with 1:1 acetone and SPI‐CHEM resin. After infiltration, samples were embedded and polymerized. Ultrathin 70‐nm thick sections were cut with diamond knives using an Ultramicrotome Leica EM UC7 (Leica, Wetzlar, Germany). The sections were collected on 200 mesh transmission electron microscopy (TEM) copper grids and counterstained with uranyl acetate and lead citrate. The ultrastructure of the sample was observed and photographed using a Tecnai G2 Spirit transmission electron microscope (FEI, Oregon) at 80 kV.

### 2.6 Western blot analysis

Sperm samples from the patient and control subject were prepared as described previously (Martinez‐Heredia, Estanyol, Ballesca, & Oliva, 2006). Each sample was centrifuged at 800 *g* for 20 min at 4°C in a 50% step Percoll gradient to remove the seminal plasma and other potentially contaminating cells in the semen. Spermatozoa protein was extracted as per the method by Liu et al., (2015), separated by 6% (w/v) SDS‐PAGE for EIF4G1 protein and 10% (w/v) SDS‐PAGE for acetylated tubulin, and then transferred to a polyvinylidene difluoride membrane (Millipore, USA). The membrane was blocked for 1 hr at room temperature (RT) with 5% skimmed milk in Tris‐buffered saline solution (pH 7.4) containing 0.05% Tween‐20 (TBST) and then incubated with either rabbit anti‐EIF4G1 (15704‐1‐AP, Proteintech, USA), anti‐COXIV (11242‐1‐AP, Proteintech, USA), anti‐ATP6 (55313‐1‐AP, Proteintech, USA), or anti‐acetylated tubulin (66200‐1‐Ig, Proteintech, USA) primary antibody overnight at 4°C. After three washes with TBST, the membranes were incubated with goat anti‐rabbit IgG secondary antibody, HRP (31460, Thermo Fisher, USA) or goat anti‐mouse IgG secondary antibody, HRP (31430, Thermo Fisher, USA) for 1 hr and washed three times with TBST at RT. The signals were developed using an ECL (enhanced chemiluminescence) kit (K‐12045‐D50, Advansta, USA) and visualized and recorded with the use of an ImageQuant LAS 4000 mini machine (GE Healthcare Life Sciences, USA). The specific antibodies used for the Western blots analysis are listed in Table S2.

### 2.7 Immunostaining of spermatozoa

Immunostaining of the spermatozoa was performed as described previously (Sha, Xu, et al., 2017). Briefly, the prepared spermatozoa were smeared onto poly‐L‐lysine coated slides, allowed to air‐dry, washed in phosphate‐buffered saline (PBS), fixed in 4% PFA (F8775, Sigma, United States) for 10 min at RT, and washed twice in PBS, followed by permeabilization with 0.2% Triton X‐100 (93443, Sigma, USA). Then, the samples were blocked with PBS containing 1% bovine serum albumin (A1933, Sigma, USA) and 2% normal goat serum (NS02L, Millipore, USA) for 30 min at RT. Slides were incubated with rabbit anti‐EIF4G1 (15704‐1‐AP, Proteintech, USA), rabbit anti‐COXIV (11242‐1‐AP, Proteintech, USA), or rabbit anti‐ATP6 (55313‐1‐AP, Proteintech, USA) primary antibodies and costained with anti‐acetylated tubulin (66200‐1‐Ig, Proteintech, USA) primary antibodies 1 hr at RT, followed by incubation with Alexa Fluor^®^ 594‐conjugated goat anti‐rabbit IgG (ZF‐0516, zsbio, China) and Alexa Fluor^®^ 488‐conjugated goat anti‐mouse IgG (ZF‐0512, zsbio, China) secondary antibodies for 45 min at RT. Slides were subsequently washed three times in PBS, mounted with Vectashield containing DAPI (Vector Laboratories) and examined under a laser scanning confocal immunofluorescence microscope (LSM510 Exiter, Carl Zeiss, Germany). The specific antibodies used in this assay are listed in Table S2.

### 2.8 Intracytoplasmic sperm injection, embryo transfer, and pregnancy follow‐up

Intracytoplasmic sperm injection (ICSI) was performed as previously described (Sha, Zhang, Ding, & Li, 2017). The couple chose to use a long‐acting GnRHa protocol. In brief, long‐acting GnRHa was injected subcutaneously on day 23 of the menstrual cycle. On the 5th day of menstruation, blood hormones levels were measured, and ultrasound imaging was performed to confirm the success of the protocol. Afterward, based on the ovarian reserve function, 150–300 U of Gn was injected daily until at least 1–2 follicles had developed to 18–20 mm and until the E2 level reached >200 pg/ml. Then, 5,000–10,000 U of HCG was injected subcutaneously at night at 10 o'clock, and eggs were retrieved 36 hr later. ICSI was performed for the couple, and fertilization was assessed 18–19 hr later for the presence of two pronuclei and two polar bodies. The fertilized oocytes were cultured individually in G1 medium (Vitrolife) until the day of transfer. Cleaved embryos were evaluated for cell number, blastomere appearance and fragmentation rate. The best embryos were selected for transfer, and extra good‐quality embryos were frozen. Two embryos were transferred by ultrasound guidance. The patient received luteal support, including 600 mg of vaginally administered micronized progesterone (Utrogestan; Besins Laboratories). Serum hCG levels were measured 14 days after embryo transfer. Clinical pregnancy was defined as a visible sac with a fetal heart beat 7 weeks after embryo transfer.

## 3 RESULTS

### 3.1 Morphological defects in the sperm of the patient with severe asthenozoospermia

**Figure 1:**
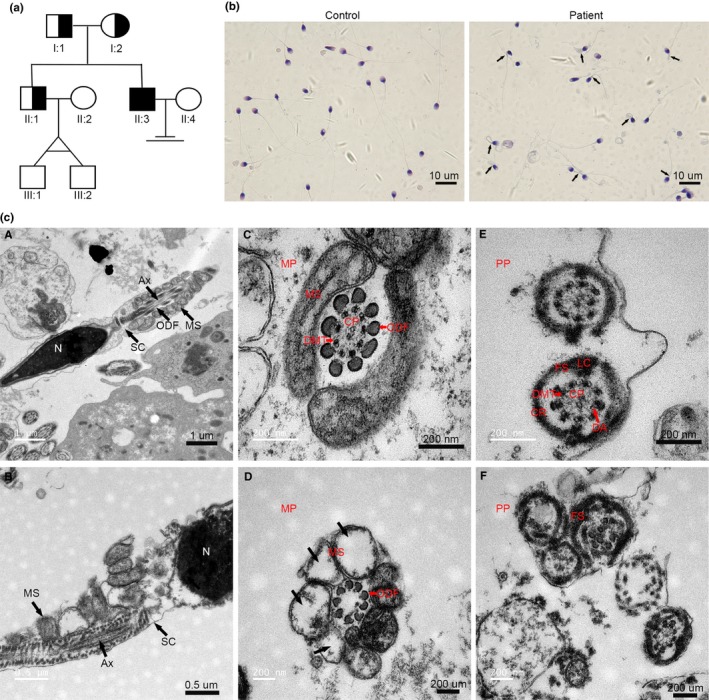
Morphological analysis in the sperm of the patient with severe asthenozoospermia (a) Family tree of the patient with severe asthenozoospermia. The black square represents the proband (II:3). (b) Morphological analysis of the sperm of patient by Papanicolaou staining. The black arrows indicate the abnormal sperm. Multiple images were taken, and representative images are presented. Scale bar: 10 μm. (c) Electron microscopic morphology of the sperm of control and the patient. (A) Longitudinal sections of sperm flagellum from the control subject. (B) Longitudinal sections of the sperm of the patient, showing damaged mitochondria and a disordered mitochondrial sheath. (C) Cross‐sections of the midpiece (MP) in the control sperm. (D) Cross‐sections of the MP in the patient's sperm. (E) Cross‐sections of the sperm flagellum in the PP of the control. (F) Cross‐sections of the sperm flagellum in the PP of the patient's sperm. Multiple images were taken, and representative images are presented. Scale bar: A = 1 μm, B = 0.5 μm, C‐F = 200 μm. Abbreviations: Ax, axoneme; CP, central pair; CR, circumferential rib; DA, dynein arm; DMT, doublet microtubule; FS, fibrous sheath; LC, longitudinal column; MP, midpiece; MS, mitochondrial sheath; N, nucleus; ODF, outer dense fiber; PP, principal piece; SC, segmented column.

The proband and his family were recruited for this study (Figure 1A). The patient's elder brother (II:1) had two sons (III:1 and III:2). The spermatozoa of the patient had low motility, and the percentages of progressive sperm and nonprogressive sperm were 0.2% and 1.1%, respectively. Therefore, he was diagnosed with severe asthenozoospermia.

As indicated by the black arrow, the sperm from the patient showed defects in the mitochondrial sheath (MS) and flagellum compared to the normal sperm by Papanicolaou staining (Figure 1B). To further characterize these defects, TEM was performed, and spermatozoa from the patient showed numerous ultrastructural defects in the MS and flagellum (Figure 1C). As indicated by the arrow, control sperm had a normal ultrastructure in the longitudinal section in the midpiece (MP) (Figure 1C, a). However, in the patient's sperm, the flagellum appeared severely disorganized, and the MS of the spermatozoon was incomplete, misassembled and much shorter than that of the normal MS (Figure 1C, b). The ultrastructure of the cross‐section showed the typical ‘“9 + 2”’ microtubule structure surrounded by a well‐organized MS in the normal spermatozoon from the control (Figure 1C, c). However, the sperm flagellum from the patient showed a complete absence of central pairs (CPs) and doublet microtubules (DMTs), mitochondria were vacuolated, and the MS showed defective assembly (Figure 1C, d). Clearly, the CPs were surrounded by nine DMTs, and the FS was composed of two longitudinal columns (LCs) connected by circumferential ribs (CR) in cross‐sections of the principal piece (PP) from the control (Figure 1C, e). In contrast, cross‐sections of sperm flagellum from the patient showed disorder. The FS was dysplastic, thickened, and dramatically disorganized. Various axonemal anomalies were observed, including a lack of CPs and DMTs (Figure 1C, f). These results show that the infertility of the patient was mainly due to vacuolated mitochondria, a short misarranged MS and an abnormal flagellum.

### 3.2 Biallelic mutations of the EIF4G1 gene were identified in the patient with severe asthenozoospermia

**Figure 2:**
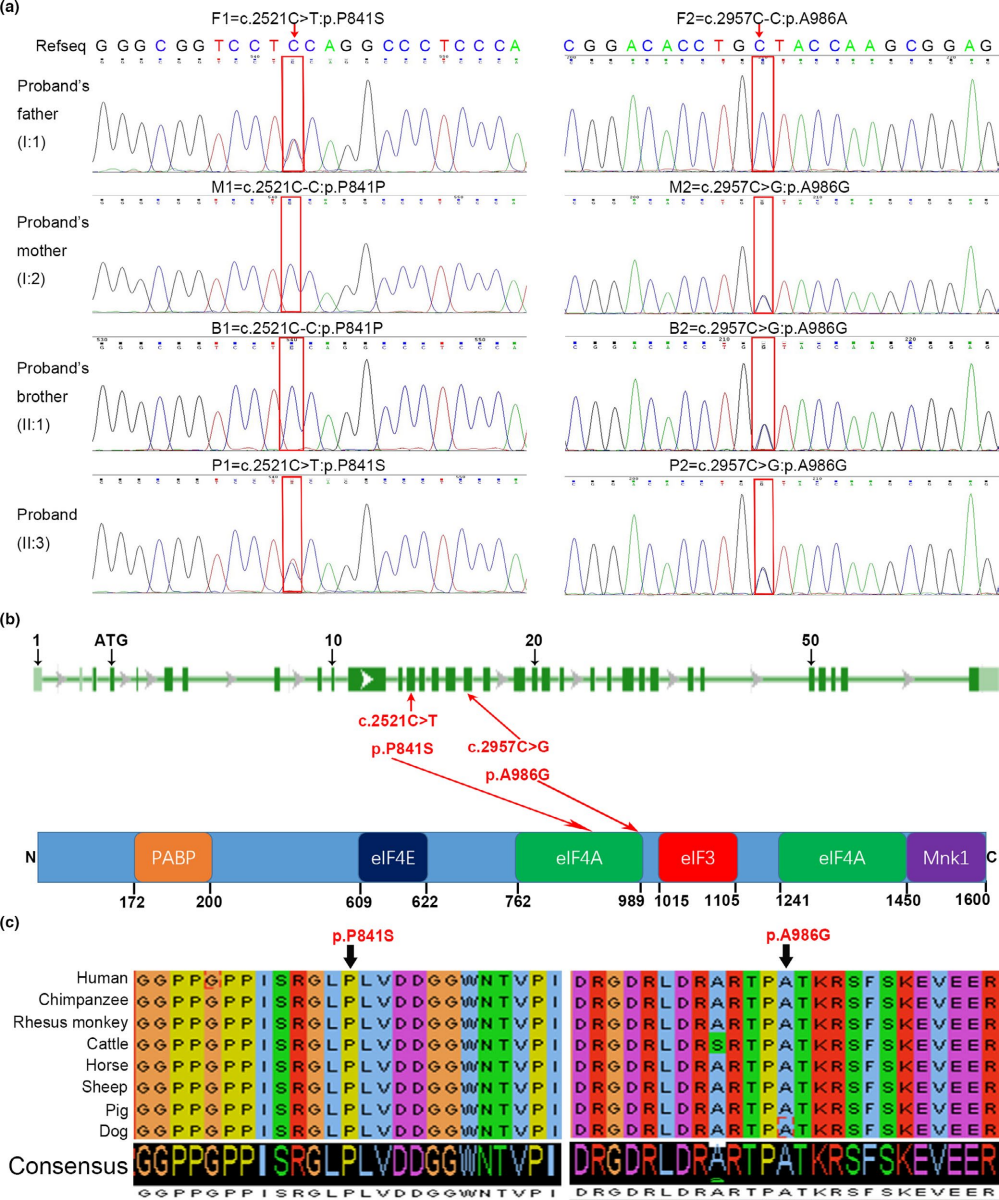
Biallelic mutations in *EIF4G1* identified in the patient with severe asthenozoospermia (a) Sanger sequencing confirmed the biallelic mutations in the *EIF4G1* gene of the proband. The red rectangle indicates the mutation sites. (b) The locations of the biallelic mutation sites in *EIF4G1*. (Top) Genomic structure and (bottom) protein domains. Green boxes indicate coding exons, and rectangles filled with color represent the functional domains. (c) Conservative analysis of the amino acids encoded by the biallelic mutation sites in different species

To determine the origin of this disease in the patient, genomic DNA was extracted from the whole blood of the patient, his elder brother and his parents, and the patient's genomic DNA was subjected to WES analysis. The results were analyzed by bioinformatics to exclude irrelevant or meaningless mutations. We obtained a list of rare and potentially pathogenic variants, including homozygous or compound heterozygous mutations (Table S3). We did not find any known genetic mutations that could cause asthenospermia on this list. After ruling out the mutations that show extremely low expression in the testis or that have low pathogenicity based on protein expression databases (https://www.proteinatlas.org) and bioinformatic analysis, we obtained biallelic mutations of *EIF4G1* (RefSeq NM_004953.4, genome build GRCh37/hg19) because this gene was the only gene that was potentially closely associated with severe asthenozoospermia based on the previous literature. A second round of Sanger sequencing for validation of the biallelic mutations in the *EIF4G1* gene was performed in the patient, his elder brother and his parents (Figure 2a). Specifically, the patient carried the following mutations: c.2521C>T: p.(Pro841Ser) (NC_000003.11: g.184043412C>T) in exon13 and c.2957C>G: p.(Ala986Gly) (NC_000003.11: g.184045117C>G) in exon17 (Figure 2a, line 3). Furthermore, we found c.2521C>T: p.(Pro841Ser) in exon13 mutation in his father (Figure 2a, line 1, left), c.2957C>G: p.(Ala986Gly) in exon17 mutation in his mother (Figure 2a, line 2, right) and c.2957C>G: p.(Ala986Gly) in exon17 mutation in his elder brother (Figure 2a, line 3, right), implying that the biallelic mutations were inherited from his parents. The biallelic mutation sites are located in the coding exons (Figure 2b, upper), and the amino acids influenced by these mutations are in the functional domains of the EIF4A‐binding sites (Figure 2b, under). In addition, the amino acids encoded by mutation sites are highly conserved between different species (Figure 2c). Furthermore, the impact of these mutations on protein function was confirmed by bioinformatics analysis using Mutation Taster databases. The mutations were highly pathogenic. The frequencies of the observed mutations in the general population were assessed using ExAC and 1,000 Genomes databases, and these mutations are rare mutation sites (Table 1).

**Table 1:** In silico analysis of *EIF4G1* mutations

Mutation	Amino acid change	Mutation taster^a^	ExAC_(total)^b^	ExAC_EA^c^	1000G_ALL^d^	1000G_EA^e^
c.2521C>T	p.P841S	Disease causing (1)	0.0001	0.0016	NA	NA
c.2957C>G	p.A986G	Disease causing (1)	0.0002	NA	0.0004	0.002

a Mutation taster (http://www.mutationtaster.org/). The probability value is the probability of the prediction, that is, a value close to 1 indicates a high “security” of the prediction.

b Frequency of variation in total of ExAC database.

c Frequency of variation in East Asian population of ExAC database.

d Frequency of variation in total of 1,000 Genomes database (A Deep Catalog of Human Genetic Variation).

e Frequency of variation in East Asian population of 1,000 Genomes database.

### 3.3 EIF4G1 protein was absent in the patient with severe asthenozoospermia

**Figure 3:**
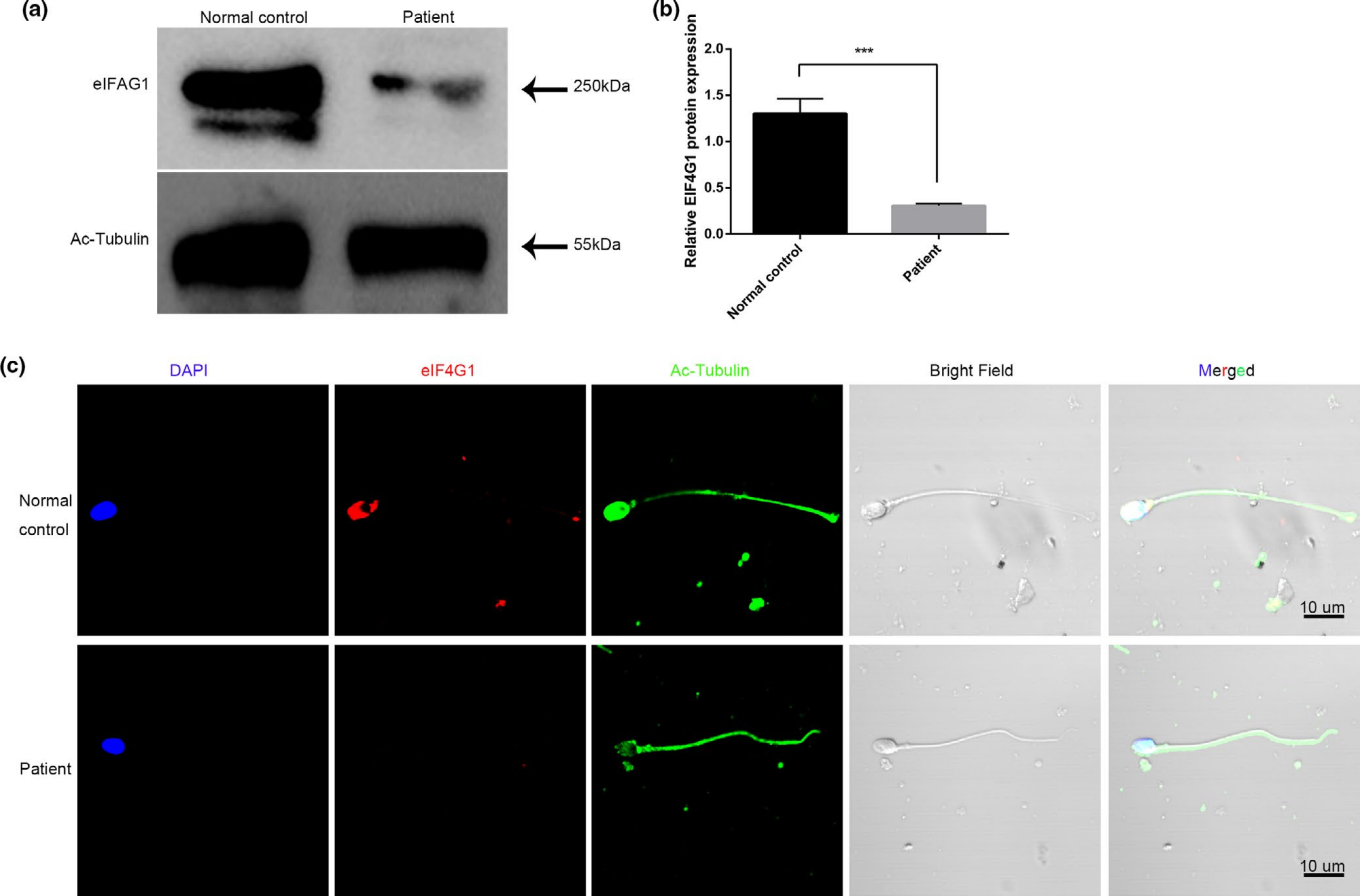
EIF4G1 protein level in the patient and control. (a) EIF4G1 protein levels were determined with Western blots. (b) The density of each band was quantified with ImageJ. Ac‐tubulin was used as the loading control. The results were expressed as the mean ± *SD* of three independent experiments. Data were analyzed with spss 18.0 software. ***p < 0.001. (c) EIF4G1 protein expression was determined with an immunofluorescence assay. Multiple images were taken, and representative images are presented. Scale bar: 10 μm.

To assess the effects of the biallelic mutations on the EIF4G1 protein, we measured the EIF4G1 protein levels in the sperm of the patient by Western blotting and immunofluorescence (Figure 3). The results showed that the expression of EIF4G1 protein in the sperm of the patient was very weak compared with that in the sperm of the control (Figure 3a). The quantification of the Western blotting results showed that protein expression was decreased significantly in the patient's sperm (Figure 3b). The protein expression level and localization of EIF4G1 were examined by immunofluorescence. EIF4G1 protein was mainly located in the head of the normal sperm, and the signal was very weak and barely detectable in the patient's sperm compared to the normal sperm (Figure 3c).

### 3.4 COXIV and ATP6 protein levels were decreased in the patient with the EIF4G1 mutations

**Figure 4:**
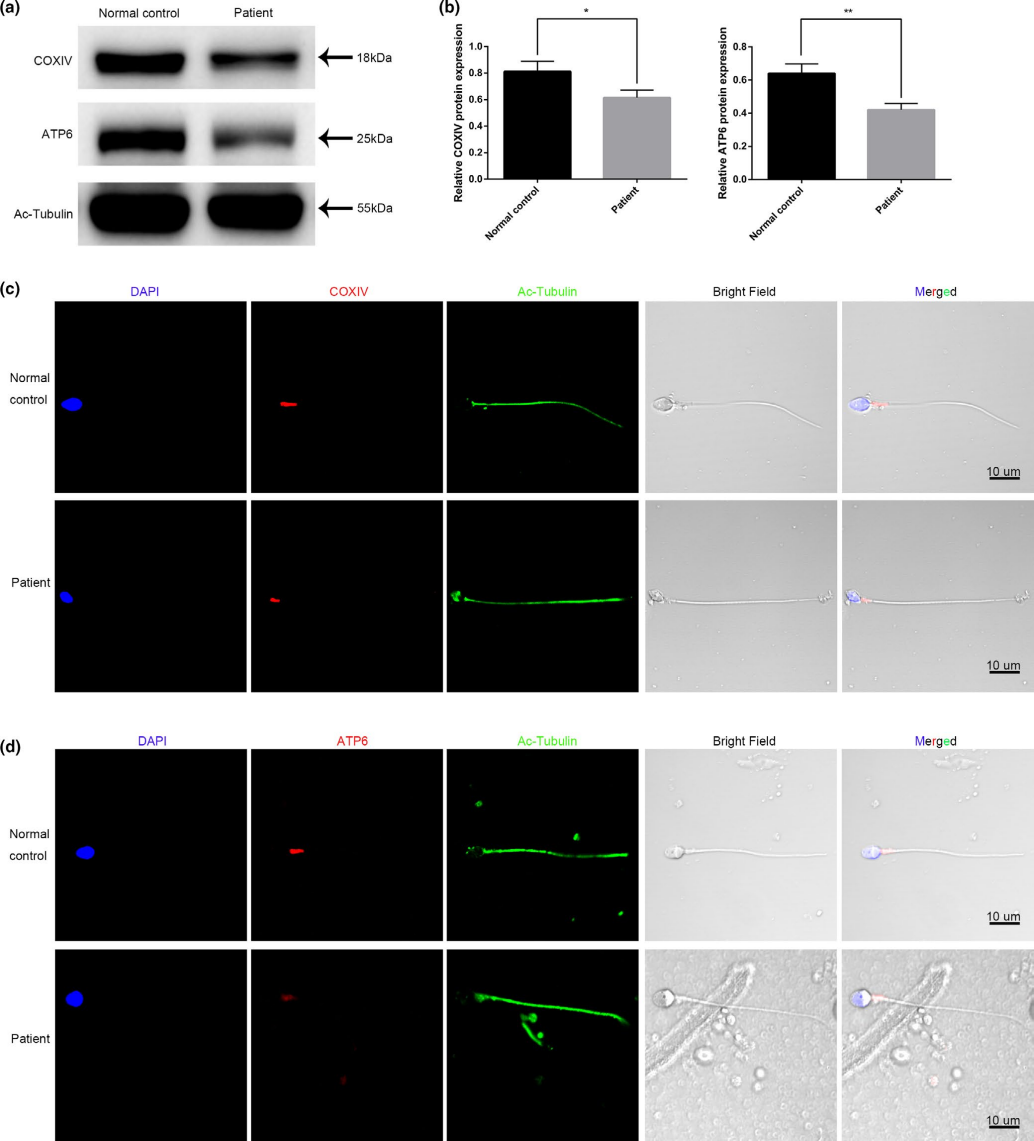
COXIV and ATP6 protein levels in the patient and control. (a) COXIV and ATP6 protein levels were determined with Western blots. (b) The density of each band was quantified with ImageJ. Ac‐tubulin was used as the loading control. The results were expressed as the mean ± *SD* of three independent experiments. Data were analyzed with spss 18.0 software. *p < 0.05, **p < 0.01. (c) COXIV and ATP6 protein expressions were determined by immunofluorescence assay. Multiple images were taken, and representative images are presented. Scale bar: 10 μm

The MS of the sperm from the patient was severely destroyed by TEM analysis. Therefore, we examined the mitochondrial marker COXIV and ATP synthesis enzyme ATP6 protein levels in the sperm of the patient by Western blotting and immunofluorescence. Western blotting showed that the expression of COXIV was decreased, and that of the ATP6 protein was decreased significantly in the sperm of the patient (Figure 4a). Quantification analysis of the Western blot band signal further confirmed the results (Figure 4b). Consistently, the immunofluorescence data also displayed the descending expression range of COXIV (Figure 4c) and the sharply decreased expression of ATP6 in the mutant sperm compared with that in the control sperm (Figure 4d).

### 3.5 Pregnancy outcome by ICSI with the patient's sperm

Assisted fertilization by ICSI was performed for this patient. The patient's wife underwent a long‐acting GnRHa protocol in our hospital in December 2017. Fifteen eggs were collected from the female, and all of them were at the MII stage. After ICSI, 11 of the fertilized eggs reached 2PN, and four of the D3 embryos, including two high‐quality embryos, were frozen. The other four embryos were grown to blastocysts (Figure S1), but the transfer was canceled due to endometrial problems. In March 2018, two high‐quality D3 embryos were transferred, but the patient's wife failed to become pregnant. Two months later, two more D3 embryos were transferred. The hCG level reached 411.27 mIU/ml in the blood 10 days after transfer, and the woman was pregnant clinically in the end.

## 4 DISCUSSION

Our present study identified biallelic mutations in the *EIF4G1* gene of a patient with severe asthenozoospermia, in which the EIF4G1 protein level was significantly decreased, the spermatozoa had lost their motility and the spermatozoa showed a variety of morphological and ultrastructural defects. These findings revealed that *EIF4G1* is a novel candidate gene associated with severe asthenozoospermia.

EIF4G1 is a component of eukaryotic initiation factor 4F (eIF4F), which is critical for recognizing the mRNA cap, unwinding the 5'‐terminal secondary structure and recruiting the mRNA to the ribosome (Aitken & Lorsch, 2012; Merrick, 2015). EIF4G1, a scaffold protein, is mainly expressed in the cytoplasm and activates translation initiation by binding eIF4E, eIF4A, eIF3, poly(A)‐binding protein, eIF4E kinase, and Mnk (Akabayov, Akabayov, & Wagner, 2014; Korneeva, Lamphear, Hennigan, & Rhoads, 2000; Zakowicz et al., 2005). Depletion of initiation factor eIF4G1 can selectively inhibit translation of specific mRNAs, but this depletion only modestly reduces overall protein synthesis in cells and impairs cell proliferation, bioenergetics, and mitochondrial activity (Ramirez‐Valle et al., 2008). In Paip2a/Paip2b‐DKO mice, flagellum formation is impaired, and the MS is absent in the middle piece. Further research proved that the expression of PABP was significantly increased and that abundant PABP competes with EIF4G1 and leads to translational inhibition (Delbes et al., 2012; Yanagiya et al., 2010). These results suggest that EIF4G1 may play an important role in spermatogenesis.

We identified biallelic mutations in *EIF4G1* (NC_000003.11:g.184043412C>T and g.184045117C>G). These changes may affect the stability of the EIF4G1 protein due to a drastic change in polarity from proline (a hydrophobic amino acid) to serine (a hydrophilic amino acid). As expected, we observed that EIF4G1 protein was significantly decreased in the sperm of the patient. Morphological analysis showed abundant defects in the MS and flagellum of the patient's sperm and that the spermatozoa lost their motility. The damaged mitochondria could not form a completed MS, which in turn damaged the structure of the sperm flagellum. In addition, we found vacuolated mitochondria in the chaotic MS and found that the mitochondrial marker COXIV and ATP synthesis enzyme ATP6 levels also decreased. This evidence suggests that these biallelic mutations may affect the function of mitochondria, leading to ultrastructural defects in the MS and flagellum.

Our findings are the first to suggest that *EIF4G1* may be a novel candidate gene that is relevant to severe asthenozoospermia. However, severe asthenozoospermia is a rare disease. Due to the limitations of sample capacity, we only found mutations in this sporadic case. A larger sample size should be investigated to further confirm the role of the *EIF4G1* mutation in severe asthenospermia. In addition, as a translation initiation factor, EIF4G1 is widely expressed and plays an important role in various tissues; thus, only the specific conditional knockout system Cre/loxP can be used to construct EIF4G1‐absent sperm in mice to validate our findings.

Hence, our study confirms that the *EIF4G1* gene mutation may be a novel pathogeny of severe asthenozoospermia and provides more information about severe asthenozoospermia for researchers and clinicians.

## CONFLICT OF INTEREST

None declared.

## ETHICAL COMPLIANCE

This study was approved by the Ethics Committees at the Xiamen Maternity and Child Care Hospital.

## References

[mgg3807-bib-0001] Aitken, C. E. , & Lorsch, J. R. (2012). A mechanistic overview of translation initiation in eukaryotes. Nature Structural & Molecular Biology, 19(6), 568–576. 10.1038/nsmb.2303 22664984

[mgg3807-bib-0002] Akabayov, S. R. , Akabayov, B. , & Wagner, G. (2014). Human translation initiation factor eIF4G1 possesses a low‐affinity ATP binding site facing the ATP‐binding cleft of eIF4A in the eIF4G/eIF4A complex. Biochemistry, 53(41), 6422–6425. 10.1021/bi500600m 25255371 PMC4204880

[mgg3807-bib-0003] Al‐Malki, A. H. , Alrabeeah, K. , Mondou, E. , Brochu‐Lafontaine, V. , Phillips, S. , & Zini, A. (2017). Testicular sperm aspiration (TESA) for infertile couples with severe or complete asthenozoospermia. Andrology, 5(2), 226–231. 10.1111/andr.12317 28187532

[mgg3807-bib-0004] Amer, M. , Metawae, B. , Hosny, H. , & Raef, A. (2013). Beneficial effect of adding pentoxifylline to processed semen samples on ICSI outcome in infertile males with mild and moderate asthenozoospermia: A randomized controlled prospective crossover study. Iranian Journal of Reproductive Medicine, 11(11), 939–944. 24639720 PMC3941394

[mgg3807-bib-0005] Amer, M. K. , Mostafa, R. M. , Fathy, A. , Saad, H. M. , & Mostafa, T. (2015). Ropporin gene expression in infertile asthenozoospermic men with varicocele before and after repair. Urology, 85(4), 805–808. 10.1016/j.urology.2014.12.033 25704993

[mgg3807-bib-0006] Amiri‐Yekta, A. , Coutton, C. , Kherraf, Z. E. , Karaouzene, T. , Le Tanno, P. , Sanati, M. H. , … Ray, P. F. (2016). Whole‐exome sequencing of familial cases of multiple morphological abnormalities of the sperm flagella (MMAF) reveals new DNAH1 mutations. Human Reproduction, 31(12), 2872–2880. 10.1093/humrep/dew262 27798045

[mgg3807-bib-0007] Baccetti, B. , Collodel, G. , Estenoz, M. , Manca, D. , Moretti, E. , & Piomboni, P. (2005). Gene deletions in an infertile man with sperm fibrous sheath dysplasia. Human Reproduction, 20(10), 2790–2794. 10.1093/humrep/dei126 15980003

[mgg3807-bib-0008] Ben Khelifa, M. , Coutton, C. , Zouari, R. , Karaouzène, T. , Rendu, J. , Bidart, M. , … Ray, P. F. (2014). Mutations in DNAH1, which encodes an inner arm heavy chain dynein, lead to male infertility from multiple morphological abnormalities of the sperm flagella. American Journal of Human Genetics, 94(1), 95–104. 10.1016/j.ajhg.2013.11.017 24360805 PMC3882734

[mgg3807-bib-0009] Byrd, M. P. , Zamora, M. , & Lloyd, R. E. (2002). Generation of multiple isoforms of eukaryotic translation initiation factor 4GI by use of alternate translation initiation codons. Molecular and Cellular Biology, 22(13), 4499–4511. 10.1128/MCB.22.13.4499-4511.2002 12052860 PMC133909

[mgg3807-bib-0010] Casper, R. F. , Meriano, J. S. , Jarvi, K. A. , Cowan, L. , & Lucato, M. L. (1996). The hypo‐osmotic swelling test for selection of viable sperm for intracytoplasmic sperm injection in men with complete asthenozoospermia. Fertility and Sterility, 65(5), 972–976. 8612859 10.1016/s0015-0282(16)58271-5

[mgg3807-bib-0011] Chartier‐Harlin, M. C. , Dachsel, J. C. , Vilariño‐Güell, C. , Lincoln, S. J. , Leprêtre, F. , Hulihan, M. M. , … Farrer, M. J. (2011). Translation initiator EIF4G1 mutations in familial Parkinson disease. American Journal of Human Genetics, 89(3), 398–406. 10.1016/j.ajhg.2011.08.009 21907011 PMC3169825

[mgg3807-bib-0012] Chemes, H. E. , Olmedo, S. B. , Carrere, C. , Oses, R. , Carizza, C. , Leisner, M. , & Blaquier, J. (1998). Ultrastructural pathology of the sperm flagellum: Association between flagellar pathology and fertility prognosis in severely asthenozoospermic men. Human Reproduction, 13(9), 2521–2526. 10.1093/humrep/13.9.2521 9806277

[mgg3807-bib-0013] Collodel, G. , Federico, M. G. , Pascarelli, N. A. , Geminiani, M. , Renieri, T. , & Moretti, E. (2011). A case of severe asthenozoospermia: A novel sperm tail defect of possible genetic origin identified by electron microscopy and immunocytochemistry. Fertility and Sterility, 95(1), 289.e11–289.e16. 10.1016/j.fertnstert.2010.05.029 20579639

[mgg3807-bib-0014] Delbes, G. , Yanagiya, A. , Sonenberg, N. , & Robaire, B. (2012). PABP interacting protein 2A (PAIP2A) regulates specific key proteins during spermiogenesis in the mouse. Biology of Reproduction, 86(3), 95 10.1095/biolreprod.111.092619 22190698

[mgg3807-bib-0015] Dimitrov, D. G. , Urbanek, V. , Zverina, J. , Madar, J. , Nouza, K. , & Kinsky, R. (1994). Correlation of asthenozoospermia with increased antisperm cell‐mediated immunity in men from infertile couples. Journal of Reproductive Immunology, 27(1), 3–12. 10.1016/0165-0378(94)90011-6 7807469

[mgg3807-bib-0016] Dirami, T. , Rode, B. , Jollivet, M. , Da Silva, N. , Escalier, D. , Gaitch, N. , … Touré, A. (2013). Missense mutations in SLC26A8, encoding a sperm‐specific activator of CFTR, are associated with human asthenozoospermia. American Journal of Human Genetics, 92(5), 760–766. 10.1016/j.ajhg.2013.03.016 23582645 PMC3644633

[mgg3807-bib-0017] Gopalkrishnan, K. , Padwal, V. , D'Souza, S. , & Shah, R. (1995). Severe asthenozoospermia: A structural and functional study. International Journal of Andrology, 18(Suppl 1), 67–74. 10.1111/j.1365-2605.1995.tb00642.x 7558392

[mgg3807-bib-0018] Harrison, R. F. (1978). Significance of sperm antibodies in human fertility. International Journal of Fertility, 23(4), 288–293. 33923

[mgg3807-bib-0019] Imataka, H. , & Sonenberg, N. (1997). Human eukaryotic translation initiation factor 4G (eIF4G) possesses two separate and independent binding sites for eIF4A. Molecular and Cellular Biology, 17(12), 6940–6947. 10.1128/MCB.17.12.6940 9372926 PMC232551

[mgg3807-bib-0020] Katsumi, M. , Ishikawa, H. , Tanaka, Y. , Saito, K. , Kobori, Y. , Okada, H. , … Miyado, M. (2014). Microhomology‐mediated microduplication in the y chromosomal azoospermia factor a region in a male with mild asthenozoospermia. Cytogenet Genome Res, 144(4), 285–289. 10.1159/000377649 25765000

[mgg3807-bib-0021] Khosronezhad, N. , Colagar, A. H. , & Jorsarayi, S. G. (2015). T26248G‐transversion mutation in exon7 of the putative methyltransferase Nsun7 gene causes a change in protein folding associated with reduced sperm motility in asthenospermic men. Reproduction, Fertility, and Development, 27(3), 471–480. 10.1071/RD13371 24384068

[mgg3807-bib-0022] Kimura, M. , Ishida, K. , Kashiwabara, S. , & Baba, T. (2009). Characterization of two cytoplasmic poly(A)‐binding proteins, PABPC1 and PABPC2, in mouse spermatogenic cells. Biology of Reproduction, 80(3), 545–554. 10.1095/biolreprod.108.072553 19020299

[mgg3807-bib-0023] Korneeva, N. L. , Lamphear, B. J. , Hennigan, F. L. , & Rhoads, R. E. (2000). Mutually cooperative binding of eukaryotic translation initiation factor (eIF) 3 and eIF4A to human eIF4G‐1. Journal of Biological Chemistry, 275(52), 41369–41376. 10.1074/jbc.M007525200 11022043

[mgg3807-bib-0024] Li, Y. S. , Feng, X. X. , Ji, X. F. , Wang, Q. X. , Gao, X. M. , Yang, X. F. , … Ma, K. (2011). Expression of SEPT4 protein in the ejaculated sperm of idiopathic asthenozoospermic men. Zhonghua Nan Ke Xue, 17(8), 699–702. 21898991

[mgg3807-bib-0025] Liu, F.‐J. , Liu, X. , Han, J.‐L. , Wang, Y.‐W. , Jin, S.‐H. , Liu, X.‐X. , … Wang, W.‐J. (2015). Aged men share the sperm protein PATE1 defect with young asthenozoospermia patients. Human Reproduction, 30(4), 861–869. 10.1093/humrep/dev003 25637620

[mgg3807-bib-0026] Lorès, P. , Coutton, C. , El Khouri, E. , Stouvenel, L. , Givelet, M. , Thomas, L. , … Touré, A. (2018). Homozygous missense mutation L673P in adenylate kinase 7 (AK7) leads to primary male infertility and multiple morphological anomalies of the flagella but not to primary ciliary dyskinesia. Human Molecular Genetics, 27(7), 1196–1211. 10.1093/hmg/ddy034 29365104

[mgg3807-bib-0027] Marchini, M. , Losa, G. , Falcone, L. , Piffaretti‐Yanez, A. , Zeeb, M. , & Balerna, M. (1991). Etiology of severe asthenozoospermia and fertility prognosis. A screening of 5216 semen analyses. Andrologia, 23(2), 115–120. 1952115 10.1111/j.1439-0272.1991.tb02513.x

[mgg3807-bib-0028] Martinez‐Heredia, J. , Estanyol, J. M. , Ballesca, J. L. , & Oliva, R. (2006). Proteomic identification of human sperm proteins. Proteomics, 6(15), 4356–4369. 10.1002/pmic.200600094 16819732

[mgg3807-bib-0029] Merrick, W. C. (2015). eIF4F: A retrospective. Journal of Biological Chemistry, 290(40), 24091–24099. 10.1074/jbc.R115.675280 26324716 PMC4591800

[mgg3807-bib-0030] Moretti, E. , Geminiani, M. , Terzuoli, G. , Renieri, T. , Pascarelli, N. , & Collodel, G. (2011). Two cases of sperm immotility: A mosaic of flagellar alterations related to dysplasia of the fibrous sheath and abnormalities of head‐neck attachment. Fertility and Sterility, 95(5), 1787.e19–1787.e23. 10.1016/j.fertnstert.2010.11.027 21144504

[mgg3807-bib-0031] Mostafa, T. , Nabil, N. , Rashed, L. , Makeen, K. , El‐Kasas, M. A. , & Mohamaed, H. A. (2018). Seminal SIRT1 expression in infertile oligoasthenoteratozoospermic men with varicocoele. Andrology, 6(2), 301–305. 10.1111/andr.12462 29359516

[mgg3807-bib-0032] Mostafa, T. , Rashed, L. , & Taymour, M. (2016). Seminal cyclooxygenase relationship with oxidative stress in infertile oligoasthenoteratozoospermic men with varicocele. Andrologia, 48(2), 137–142. 10.1111/and.12423 25906828

[mgg3807-bib-0033] Ramirez‐Valle, F. , Braunstein, S. , Zavadil, J. , Formenti, S. C. , & Schneider, R. J. (2008). eIF4GI links nutrient sensing by mTOR to cell proliferation and inhibition of autophagy. Journal of Cell Biology, 181(2), 293–307. 10.1083/jcb.200710215 18426977 PMC2315676

[mgg3807-bib-0034] Sha, Y. W. , Sha, Y. K. , Ji, Z. Y. , Mei, L. B. , Ding, L. , Zhang, Q. , … Li, L. (2018). TSGA10 is a novel candidate gene associated with acephalic spermatozoa. Clinical Genetics, 93(4), 776–783. 10.1111/cge.13140 28905369

[mgg3807-bib-0035] Sha, Y. W. , Xu, X. , Mei, L. B. , Li, P. , Su, Z. Y. , He, X. Q. , & Li, L. (2017). A homozygous CEP135 mutation is associated with multiple morphological abnormalities of the sperm flagella (MMAF). Gene, 633, 48–53. 10.1016/j.gene.2017.08.033 28866084

[mgg3807-bib-0036] Sha, Y. , Yang, X. , Mei, L. , Ji, Z. , Wang, X. U. , Ding, L. U. , … Yang, S. (2017). DNAH1 gene mutations and their potential association with dysplasia of the sperm fibrous sheath and infertility in the Han Chinese population. Fertility and Sterility, 107(6), 1312–1318 e1312. 10.1016/j.fertnstert.2017.04.007 28577616

[mgg3807-bib-0037] Sha, Y. W. , Zhang, Q. , Ding, L. , & Li, P. (2017). First successful pregnancy outcome after intracytoplasmic sperm injection with short‐tailed sperm from an infertile Han Chinese man. Asian Journal of Andrology, 19(5), 613–614. 10.4103/1008-682X.182395 27427550 PMC5566859

[mgg3807-bib-0038] Shibahara, H. , Shiraishi, Y. , & Suzuki, M. (2005). Diagnosis and treatment of immunologically infertile males with antisperm antibodies. Reproductive Medicine and Biology, 4(2), 133–141. 10.1111/j.1447-0578.2005.00102.x 29699216 PMC5906991

[mgg3807-bib-0039] Takasaki, N. , Tachibana, K. , Ogasawara, S. , Matsuzaki, H. , Hagiuda, J. , Ishikawa, H. , … Narimatsu, H. (2014). A heterozygous mutation of GALNTL5 affects male infertility with impairment of sperm motility. Proceedings of the National Academy of Sciences, 111(3), 1120–1125. 10.1073/pnas.1310777111 PMC3903224 24398516

[mgg3807-bib-0040] Wang, X. , Jin, H. , Han, F. , Cui, Y. , Chen, J. , Yang, C. , … Gao, Z. (2017). Homozygous DNAH1 frameshift mutation causes multiple morphological anomalies of the sperm flagella in Chinese. Clinical Genetics, 91(2), 313–321. 10.1111/cge.12857 27573432

[mgg3807-bib-0041] Wilton, L. J. , Temple‐Smith, P. D. , & de Kretser, D. M. (1992). Quantitative ultrastructural analysis of sperm tails reveals flagellar defects associated with persistent asthenozoospermia. Human Reproduction, 7(4), 510–516. 10.1093/oxfordjournals.humrep.a137681 1522195

[mgg3807-bib-0042] Xu, X. , Sha, Y. W. , Mei, L. B. , Ji, Z. Y. , Qiu, P. P. , Ji, H. , … Li, L. (2018). A familial study of twins with severe asthenozoospermia identified a homozygous SPAG17 mutation by whole‐exome sequencing. Clinical Genetics, 93(2), 345–349. 10.1111/cge.13059 28548327

[mgg3807-bib-0043] Yan, R. , & Rhoads, R. E. (1995). Human protein synthesis initiation factor eIF‐4 gamma is encoded by a single gene (EIF4G) that maps to chromosome 3q27‐qter. Genomics, 26(2), 394–398. 7601469 10.1016/0888-7543(95)80227-d

[mgg3807-bib-0044] Yanagiya, A. , Delbes, G. , Svitkin, Y. V. , Robaire, B. , & Sonenberg, N. (2010). The poly(A)‐binding protein partner Paip2a controls translation during late spermiogenesis in mice. J Clin Invest, 120(9), 3389–3400. 10.1172/JCI43350 20739757 PMC2929737

[mgg3807-bib-0045] Zakowicz, H. , Yang, H. S. , Stark, C. , Wlodawer, A. , Laronde‐Leblanc, N. , & Colburn, N. H. (2005). Mutational analysis of the DEAD‐box RNA helicase eIF4AII characterizes its interaction with transformation suppressor Pdcd4 and eIF4GI. RNA, 11(3), 261–274. 10.1261/rna.7191905 15661843 PMC1370716

[mgg3807-bib-0046] Zhang, Y. , Malekpour, M. , Al‐Madani, N. , Kahrizi, K. , Zanganeh, M. , Mohseni, M. , … Smith, R. J. H. (2007). Sensorineural deafness and male infertility: A contiguous gene deletion syndrome. Journal of Medical Genetics, 44(4), 233–240. 10.1136/jmg.2006.045765 17098888 PMC2598039

