# Supplementary figures and images for: EIF4G1 is a novel candidate gene associated with severe asthenozoospermia
Source: Mol Genet Genomic Med. 2019 Jul 3;7(8):e807. doi: 10.1002/mgg3.807 (PMC6687618; doi:10.1002/mgg3.807)

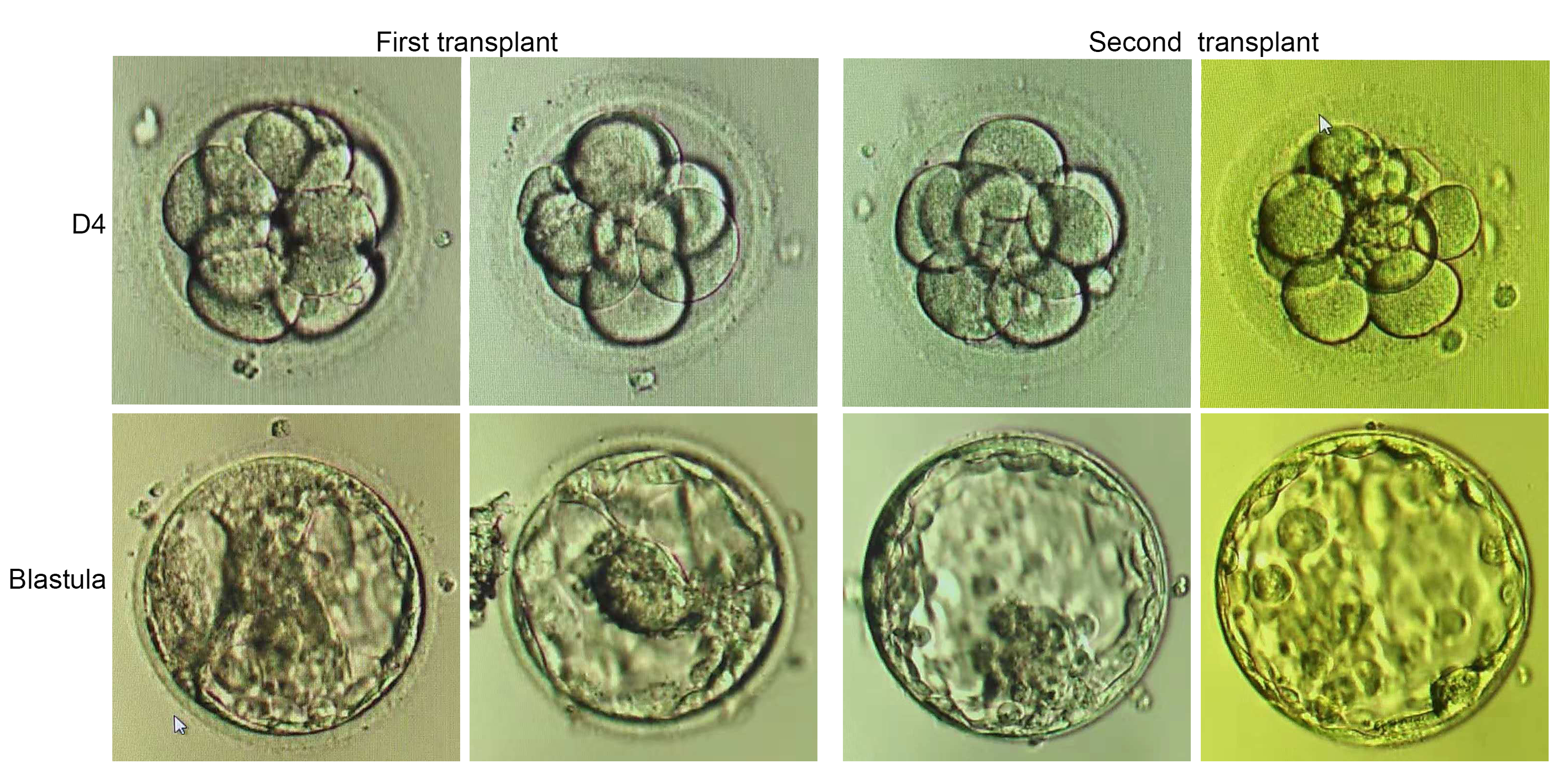

Supplement: Supplementary file 1 [file MGG3-7-e807-s001.tif]
