# Supplementary material for: EIF4G1 is a novel candidate gene associated with severe asthenozoospermia
Source: Mol Genet Genomic Med. 2019 Jul 3;7(8):e807. doi: 10.1002/mgg3.807 (PMC6687618; doi:10.1002/mgg3.807)
Supplement: Supplementary file 2 [file MGG3-7-e807-s002.docx]

**Supplemental Table 1: *EIF4G1* primers used for sanger sequencing**

| Target | Forward | Reverse |
| --- | --- | --- |
| exon13:c.2521C>T | TTGAGTGCCTTTGTCGTCTG | GGTGACTCTGTGGTACTACC |
| exon17:c.2957C>G | TTGGGTTAGATTGGGGCATA | GGAAAGGGAGGTAAGGAGAG |

**Supplementary Table 2: Antibodies used in Western blot and immunofluorescence.**

| Primary/ Secondary antibodies | Host species | Company | Cat no. | Application | Dilution |
| --- | --- | --- | --- | --- | --- |
| EIF4G1 | Rabbit | Proteintech | 15704-1-AP | WB | 1:1000 |
|  |  |  |  | IF | 1:50 |
| COXIV | Rabbit | Proteintech | 11242-1-AP | WB | 1:4000 |
|  |  |  |  | IF | 1:100 |
| ATP6 | Rabbit | Proteintech | 55313-1-AP | WB | 1:500 |
|  |  |  |  | IF | 1:100 |
| Acetylated Tubulin | Mouse | Proteintech | 66200-1-Ig | WB | 1:3000 |
|  |  |  |  | IF | 1:1000 |
| Goat anti-Rabbit IgG (H+L) Secondary Antibody, HRP | Goat | ThermoFisher | 31460 | WB | 1:5000 |
| Goat anti-Mouse IgG (H+L) Secondary Antibody, HRP | Goat | Thermofisher | 31430 | WB | 1:5000 |
| Alexa Fluor® 488 - Conjugated Goat anti-Mouse IgG（H+L） | Goat | ZSGB-BIO | ZF-0512 | IF | 1:200 |
| Alexa Fluor® 594 - Conjugated Goat anti-Rabbit IgG（H+L） | Goat | ZSGB-BIO | ZF-0516 | IF | 1:200 |

**Supplementary Table 3: The rare and potential pathnogenic mutatinos in the patient with severe asthenozoospermia**

| **Gene** | **Change** | **Annotation** |  |  |  |
| --- | --- | --- | --- | --- | --- |
| AGAP3 | frameshift insertion | AGAP3:NM_001042535:exon1:c.94_95insGGGG:p.C32fs |  |  |  |
| ARSD | nonsynonymous SNV | ARSD:NM_001669:exon5:c.713G>T:p.C238F |  |  |  |
| ARSD | nonsynonymous SNV | ARSD:NM_001669:exon5:c.719T>G:p.F240C |  |  |  |
| C6orf223 | nonframeshift insertion | C6orf223:NM_153246:exon4:c.369_370insGCGGCG:p.R123delinsRAA |  |  |  |
| CLIC2 | nonsynonymous SNV | CLIC2:NM_001289:exon5:c.502G>A:p.V168I |  |  |  |
| COL27A1 | nonsynonymous SNV | COL27A1:NM_032888:exon1:c.20G>T:p.R7L |  |  |  |
| COL27A1 | nonsynonymous SNV | COL27A1:NM_032888:exon8:c.2159C>T:p.P720L |  |  |  |
| **EIF4G1** | **nonsynonymous SNV** | **EIF4G1:NM_004953:exon13:c.2521C>T:p.P841S** |  |  |  |
| **EIF4G1** | **nonsynonymous SNV** | **EIF4G1:NM_004953:exon17:c.2957C>G:p.A986G** |  |  |  |
| MADCAM1 | nonframeshift insertion | MADCAM1:NM_130760:exon4:c.742_743insCTCCCGA  CACCACCTCCCAGGAGCCTCCCGACACCACCTCCC  AGGAGC:p.S248delinsSPDTTSQEPPDTTSQEP |  |  |  |
| MADCAM1 | nonframeshift insertion | MADCAM1:NM_130760:exon4:c.681_682insACCTCCCC  GGAGCCTCCCGACACC:p.P227delinsPTSPEPPDT |  |  |  |
| MUC3A | nonsynonymous SNV | MUC3A:NM_005960:exon1:c.22G>A:p.G8S |  |  |  |
| MUC3A | nonsynonymous SNV | MUC3A:NM_005960:exon1:c.25C>A:p.L9I |  |  |  |
| MYOCD | nonsynonymous SNV | MYOCD:NM_153604:exon11:c.2081A>G:p.D694G |  |  |  |
| MYOCD | nonsynonymous SNV | MYOCD:NM_153604:exon13:c.2376C>A:p.S792R |  |  |  |
| NOC4L | nonsynonymous SNV | NOC4L:NM_024078:exon12:c.1187G>A:p.R396H |  |  |  |
| NOC4L | frameshift deletion | NOC4L:NM_024078:exon9:c.897_901del:p.D299fs |  |  |  |
| PER3 | nonsynonymous SNV | PER3:NM_001289861:exon18:c.2780C>T:p.S927L |  |  |  |
| PER3 | nonsynonymous SNV | PER3:NM_001289861:exon14:c.1555A>G:p.T519A |  |  |  |
| SAAL1 | nonframeshift insertion | SAAL1:NM_138421:exon1:c.58_59insAGGAGG:p.V20delinsEEV |  |  |  |
| SPATA31A1 | nonsynonymous SNV | SPATA31A1:NM_001085452:exon4:c.3338G>A:p.S1113N |  |  |  |
| SSC5D | frameshift deletion | SSC5D:NM_001144950:exon14:c.3807delG:p.M1269fs |  |  |  |
| SSC5D | frameshift deletion | SSC5D:NM_001144950:exon14:c.3810delG:p.Q1270fs |  |  |  |
| TRIM64B | nonsynonymous SNV | TRIM64B:NM_001164397:exon1:c.202C>G:p.L68V |  |  |  |
| TTN | nonsynonymous SNV | TTN:NM_003319:exon158:c.61026A>T:p.E20342D |  |  |  |
| TTN | nonsynonymous SNV | TTN:NM_003319:exon154:c.45797G>A:p.R15266H |  |  |  |
| ZNF469 | nonsynonymous SNV | ZNF469:NM_001127464:exon2:c.10633G>A:p.G3545R |  |  |  |
| ZNF469 | nonsynonymous SNV | ZNF469:NM_001127464:exon2:c.4423C>G:p.L1475V |  |  |  |
| ZNF83 | nonframeshift deletion | ZNF83:NM_001277951:exon3:c.801_884del:p.267_295del |  |  |  |
